# Optimized BERT-based NLP outperforms zero-shot methods for automated symptom detection in clinical practice

**DOI:** 10.3389/fdgth.2025.1623922

**Published:** 2025-11-26

**Authors:** Juan G. Diaz Ochoa, Natalie Layer, Jonas Mahr, Faizan E Mustafa, Christian U. Menzel, Martina Müller, Tobias Schilling, Gerald Illerhaus, Markus Knott, Alexander Krohn

**Affiliations:** 1 QuiBiQ GmbH, Stuttgart, Germany; 2 PerMediQ GmbH, Stuttgart, Germany; 3 Klinikum Stuttgart, Stuttgart Cancer Center - Tumorzentrum Eva Mayr-Stihl DE, Stuttgart, Germany; 4 Department for Emergency and Intensive Care Medicine (DIANI), Klinikum Stuttgart, Stuttgart, Germany; 5 Department of Internal Medicine I, University Hospital Regensburg, Regensburg, Germany

**Keywords:** natural language processing (NLP), named entity recognition (NER), symptom extraction, large language models (LLM), fine-tuning, clinical NLP

## Abstract

**Background:**

Large Language Models (LLMs) have raised broad expectations for clinical use, particularly in the processing of complex medical narratives. However, in practice, more targeted Natural Language Processing (NLP) approaches may offer higher precision and feasibility for symptom extraction from real-world clinical texts. NLP provides promising tools for extracting clinical information from unstructured medical narratives. However, few studies have focused on integrating symptom information from free texts in German, particularly for complex patient groups such as emergency department (ED) patients. The ED setting presents specific challenges: high documentation pressure, heterogeneous language styles, and the need for secure, locally deployable models due to strict data protection regulations. Furthermore, German remains a low-resource language in clinical NLP.

**Methods:**

We implemented and compared two models for zero-shot learning—GLiNER and Mistral—and a fine-tuned BERT-based SCAI-BIO/BioGottBERT model for named entity recognition (NER) of symptoms, anatomical terms, and negations in German ED anamnesis texts in an on-premises environment in a hospital. Manual annotations of 150 narratives were used for model validation. The postprocessing steps included confidence-based filtering, negation exclusion, symptom standardization, and integration with structured oncology registry data. All computations were performed on local hospital servers in an on-premises implementation to ensure full data protection compliance.

**Results:**

The fine-tuned SCAI-BIO/BioGottBERT model outperformed both zero-shot approaches, achieving an F1 score of 0.84 for symptom extraction and demonstrating superior performance in negation detection. The validated pipeline enabled systematic extraction of affirmed symptoms from ED-free text, transforming them into structured data. This method allows large-scale analysis of symptom profiles across patient populations and serves as a technical foundation for symptom-based clustering and subgroup analysis.

**Conclusions:**

Our study demonstrates that modern NLP methods can reliably extract clinical symptoms from German ED free text, even under strict data protection constraints and with limited training resources. Fine-tuned models offer a precise and practical solution for integrating unstructured narratives into clinical decision-making. This work lays the methodological foundation for a new way of systematically analyzing large patient cohorts on the basis of free-text data. Beyond symptoms, this approach can be extended to extracting diagnoses, procedures, or other clinically relevant entities. Building upon this framework, we apply network-based clustering methods (in a subsequent study) to identify clinically meaningful patient subgroups and explore sex- and age-specific patterns in symptom expression.

## 1 Introduction

Clinical notes are valuable tools for describing patients’ phenomenal experiences, such as symptoms. These experiences, or *qualia*, refer to the subjective, qualitative aspects of perception that may not always directly reflect disease processes (1). In medical practice, physicians interpret these subjective reports and translate them into structured descriptions that guide diagnosis and treatment. This interpretive process is central to clinical reasoning, as physicians’ impressions and *gestalt* influence how symptoms are prioritized and linked to potential diagnoses (2). While standardized codes in electronic health records (EHRs), such as the ICD-10 or CEDIS, capture essential information, they fail to reflect the full complexity of patients’ lived experience, underscoring the importance of free-text clinical documentation in patient care.

Analyzing these narratives is therefore essential for a comprehensive understanding of the clinical context. However, anamnesis texts are typically unstructured, manually written, and highly variable in style (3). Although they contain rich information relevant for diagnosis, decision-making, and research, their unstructured nature hinders their systematic use. Hence, methods are needed to transform these texts into structured, analyzable formats.

While conventional machine learning models process structured data efficiently, they often fall short in reproducing the nuanced, contextual understanding of experienced physicians in complex scenarios (4). To be clinically applicable, models must preserve the semantic richness of clinical narratives rather than flattening them into overly simplified representations.

NLP technologies have already been used to extract structured elements from EHRs (5), often employing transformer-based architectures such as BERT and tagging schemes such as BIO for named entity recognition (NER) (6). In emergency medicine, NER and relation extraction (RE) have proven useful for identifying clinical concepts, events, and relationships.

Similarly, LLMs (Large Language Models) have also been applied for entity extraction from medical texts and text labeling, with the first studies demonstrating high reliability in the competition of this task, even in languages different from English (7); in such studies, API (Application Programming Interface) requests to models deployed in external servers were implemented to complete this task, which implies that patient-data records are evaluated in the cloud.

However, many studies focus on highly represented languages such as English or Mandarin, with German being substantially underrepresented. German presents unique linguistic challenges, including flexible word order, compound structures and complex negation forms, which reduce model transferability and performance (8). Furthermore, in the case of LLM, the use of an API connected to external servers violates data protection rules in the hospital context.

Symptom extraction methods range from rule-based approaches to machine learning-assisted NER techniques (6). However, few studies treat symptoms as the primary target entities (9). This is especially relevant for ED texts, which are written under time pressure and thus exhibit inconsistency and syntactic noise (10). Improving model performance in this domain requires annotated corpora and real-world data integration.

Recent studies have shown that combining structured and unstructured data improves predictions of ED severity compared with using structured data alone (11), emphasizing the value of anamnesis texts beyond administrative functions. Our approach builds upon this insight and uses fine-tuned NLP models to extract symptoms from German ED records—particularly from oncology patients, who often present with polysymptomatic profiles and high clinical complexity.

Unlike conventional approaches that search for predefined symptom lists (11), we employ a holistic, data-driven method to map all affirmed symptoms directly from the narrative, reducing bias and preserving patient-centered documentation. Owing to their complex disease trajectories and the availability of high-quality registry data for retrospective validation, cancer patients serve as a testbed for this approach.

In this study, we first evaluate three NLP models—1) GLiNER, 2) Mistral-Nemo-Instruct-2407, and 3) a fine-tuned SCAI-BIO/BioGottBERT model—for their performance in symptom, negation, and anatomy recognition in German ED texts. We focus on their applicability in clinical practice, data protection compliance, and capacity to capture semantic nuance.

## 2 Methods

We analyzed emergency department data from the Klinikum Stuttgart, Germany, for patients aged 18 years and older from December 16, 2010, to September 12, 2024, totaling 256,453 ED visits. Simultaneously, the ONKOSTAR register, an SQL database for quality assurance and cancer registry reporting, contained 35,937 oncology patients within the same period.

Matching both datasets revealed 10,036 individuals with a cancer diagnosis and at least one ED visit, representing 3.9% of all ED patients. This result aligns with data from comparable emergency departments (12).

### 2.1 Annotated clinical data for model fine tunning

To fine-tune the machine learning models, we extracted 150 anamnesis texts from the emergency department database. All the texts were manually deidentified to allow annotation outside the hospital environment, ensuring compliance with data protection regulations and ethical standards. No metadata capable of identifying individual patients were stored or shared. Although the entire processing pipeline can be deployed on local hospital servers, manual deidentification serves as an additional safeguard to minimize any residual risk of patient reidentification.

Symptom expressions and relevant clinical features—such as preexisting conditions and documented procedures—were annotated manually by two independent annotators via Doccano, an open-source annotation tool for text data.^1^ In addition, we annotated anatomical references and negations. The inclusion of negations allowed us to differentiate affirmed from excluded symptoms (e.g., “no dyspnea”), whereas anatomical annotations helped localize symptoms within the body (e.g., “pain in the lower abdomen”). One annotator has a scientific background in medicine and holds a doctorate; the second annotator has a scientific background in natural sciences but has a good understanding of medicine and holds a doctorate as well. Both annotators therefore possess high competence in scientific work and are well qualified to understand and reliably classify clinical anamnesis texts.

Annotations were converted into token-level labels via the inside–outside–beginning (IOB) tagging scheme. Tokens at the start of a named entity were labeled ‘B-’, tokens inside an entity were labeled ‘I-’, and all other tokens were labeled ‘O’. This encoding enabled structured processing by the downstream NLP models. In summary, this annotated dataset provided a robust and consistent foundation for the final fine-tuning of the models.

The trained named entity recognition (NER) models were then used to extract structured information from the ED corpus. For each patient, we retained only the anonymized identifier along with basic clinical metadata (e.g., diagnosis, sex, age) and the extracted symptoms. All processing steps were executed within the hospital infrastructure, with strict adherence to data protection policies.

### 2.2 NER methods

NLP has proven effective in extracting insights from unstructured text data. Transformer-based models, a type of deep learning architecture, use self-attention mechanisms and parallel processing to analyze linguistic relationships and identify patterns in large datasets (13). These models consist of multiple layers that refine the data representation across different levels. By analyzing words in the context of entire sentences, they infer meaning and relationships more accurately. Parallel processing further enhances efficiency and speed (13).

Several studies have analyzed the performance of benchmark models applied to biomedical data, particularly in zero-shot settings^2^ (14). However, all these models have been tested on clinical texts written in English. Furthermore, these implementations focus only on relevant clinical entities, whereas other relevant entities, such as negations, are not tested. For this reason, a more exhaustive analysis is required for NER models applied to German.

We utilize BERT-based models [bidirectional encoder representations from transformers, i.e., the ability to consider both the left and right contexts of words simultaneously^3^ (15)], a widely adopted model specifically for text analysis (15). Our previous research confirmed its suitability for capturing the semantic complexity of medical narratives (16). This transformer architecture is present in both the implemented models, the GlinNER model and the SCAI-BIO/BioGottBERT-based model.

Furthermore, we implemented Mistral-Nemo-Instruct-2407 as a zero-shot LLM model in a local deployment. We selected this model because of its high performance, simplicity in implementation, customization, and, more importantly, multilingual support.^4^ In comparison to the API access to the large Mistral model (123 billion of parameters), the local implemented Mistral-Nemo-Instruct is significantly smaller (12 billion of parameters) and thus limited in its computational capabilities.

In summary, we evaluated the following three NLP models for automated symptom extraction from emergency department anamnesis texts:

- Mistral-Nemo-Instruct-2407 (Open weight transformer-based Large Language Model, LLM) Model^5^ (17) [Zero-Shot]
- GLiNER (Generalized Language Model for Information Extraction and Recognition) Model^6^^,^^7^ (18) [Zero-shot]
- SCAI-BIO/BioGottBERT-based model (Scientific Curation & Annotation Initiative – Biomedical version/Biomedical GottBERT) Model^8^ (16) [Fine-tuning]

GliNER and Mistral-Nemo-Instruct-2407 were applied in a zero-shot setting, relying on their pretrained general language understanding without task-specific adaptation. In particular, we consider in this study the Mistral-Nemo-Instruct-2407 as base line model.

The implemented prompt for the Mistral-Nemo-Instruct-2407 has been defined following the schema proposed by Ashok and Lipton (19); in this implementation the original four parts in the prompt-schema were reduced into the following three parts (see Appendix 1):

- Part 1: Definition of the entities that should be recognized in the narrative. Entities are symptoms, anatomic position and negation.
- Part 2: Few examples with solutions are provided in the prompt.
- Part 3: The model is prompted to give the reasoning behind its classification of entities and asked to provide a list, including optional candidate entities.

The prompt implemented in part 3 of the whole prompt is shown in Table 1.

**Table 1:** Example of the prompt in part 3 of the ashok-lipton prompting schema.

Original prompt in German	English version
Ermittele anhand des obigen Absatzes eine Liste möglicher Entities und erkläre, warum es sich entweder um ein Entity handelt oder nicht. Gebe alle gefundenen Entities auch in einer Python Liste wie folgt zurück, füge keine Kommentare hinzu:	Use the paragraph above (see Appendix 1) to determine a list of possible entities and explain why it is either an entity or not. Return all found entities also in a Python list like this, don't add comments:

In Appendix 1 we show the whole prompt implemented using the Ashok-Lipton schema (German as well as corresponding English translation). This prompt is then used for each clinical narrative to extract the classified entities via LLM.

In contrast to the previous two models, the SCAI-BIO/BioGottBERT model was further fine-tuned via a small-annotated dataset (see section “annotated data for model fine tuning”). Owing to its BERT-based architecture, it captures contextual dependencies and semantic nuances more effectively than traditional NLP methods such as bag-of-words or TF-IDF (term frequency-inverse document frequency) (20).

The comparison between different models enabled us to assess the reliability and applicability of zero-shot modeling vs. model fine-tuning for clinical information extraction. All the models were deployed locally on hospital servers, ensuring full compliance with data protection regulations and offering operational flexibility within clinical environments.

### 2.3 Methods to increase the precision of extracted symptoms

For symptoms identified as “Schmerzen” (pain) that were not part of a compound term [e.g., “Kopfschmerzen” (headache)], we searched for anatomical references in the preceding or following entries. If such an anatomy was found, it was combined with the symptoms to provide a more specific description (e.g., anatomy + symptoms). This allowed us to include detailed pain symptoms, such as “Kopfschmerzen”, instead of the generic “Schmerzen” (see an example in Table 2).

**Table 2:** Example of original text including both symptoms (blue) and negated symptoms (red) detected by the model [original text is in German; in this table we deploy the corresponding English translation (E)].

	Clinical narrative	Detected by model
E	4 weeks ago surgery for colon Ca with artificial exit on the right. In case of closure, resurgery with inpatient stay up to 2 weeks ago. For days significant Swelling of the right lower leg with redness and overheating. No dyspnea, no thoracic pain.	Swelling; redness; overheating

Next, we cleaned the symptom entries by standardizing their format. All symptoms were converted to lowercase, with the first letter capitalized, and punctuation, such as periods and colons, was removed. For pain-related symptoms, the anatomy was directly combined with the term, so “head + ache” (“Kopf + Schmerzen”) became headache (“Kopfschmerzen”).

To further streamline the data, we created a dictionary of common symptoms that included synonyms and word variations. This ensured that similar terms, such as “Verstopfung” and “Obstipation” (both obstipation), were standardized to a single term. We also accounted for word inflections [e.g., “husten” (cough) and “gehustet” (coughed)] to capture diverse forms of expression. Symptoms that matched the dictionary were replaced with the standardized term, whereas unmatched symptoms retained their original name.

## 3 Results

We developed and validated a fine-tuned NLP approach to extract affirmed symptoms from unstructured ED anamnesis texts. Oncology patients, a clinically complex and vulnerable group, were selected because of the availability of high-quality registry data, allowing robust retrospective validation. We transformed free text into structured symptom profiles and linked patients by symptom similarity, age group, and sex. This enabled the identification of clinically meaningful subgroups on the basis of symptom constellations.

### 3.1 Annotation and evaluation strategy

The first step in developing our NLP model involved validating its ability to identify symptoms accurately via a structured annotation and evaluation workflow. Text annotation was carried out independently by two annotators. To assess the consistency of the annotations, interannotator agreement was measured. During the annotation process, we observed occasional differences between annotators, both in terms of the selected text spans and the assigned entity types (e.g., disease vs. symptom).

To account for such variations and evaluate model performance in a structured manner, we adopted four levels of evaluation granularity, following established standards in clinical named entity recognition (NER) (21). These evaluation metrics range from a strict match—requiring exact agreement in both the text boundaries and the entity type—to more lenient metrics that tolerate partial overlaps or disregard the entity type. This allows for a more nuanced assessment of model performance (see Table 3).

**Table 3:** Different validation metrics implemented in this research**.**

Kind of validation metric	Interpretation
Strict	Exact match of both text boundaries and entity type
Exact	Exact match of text boundaries, regardless of entity type
Partial	Partial overlap of text boundaries, regardless of entity type
Entity type	Matching entity type, allowing for some overlap with the gold standard annotation

### 3.2 Interannotator agreement

To evaluate interannotator agreement, 20 narratives were annotated in parallel. While Cohen's kappa is commonly used for document-level tasks, the F1 score is better suited for entity-level evaluations, such as symptom extraction. Table 3 shows the agreement across the four levels of granularity.

The highest agreement was observed for the entity type metric, with an F1 score of 0.72, precision of 0.75, and recall of 0.69. These metrics reflect how reliably symptoms were identified: precision measures how many of the extracted symptoms were correct, recall indicates how many of the relevant symptoms were captured, and the F1 score balances both measures into a single value (see Table 4).

**Table 4:** Validation of interannotator agreement (*n* = 20 narratives).

Validation metrics	Entity type	Exact	Partial	Strict
F1	0.72	0.58	0.68	0.56
Precision	0.75	0.60	0.70	0.58
Recall	0.69	0.56	0.65	0.54

The consistent performance across all the metrics confirmed the robustness of our annotation strategy.

Nevertheless, there were some challenges with the annotation of the clinical narratives, especially with typically complex text compositions in German. Common cases were the annotation of adjectives and entities (e.g., a symptom) in a single span as well as typical German compound words, where anatomy and symptom are written in a single word. Thus, while one annotator might annotate “Rückenschmerzen” (Pain in the back) in a single span, another annotator might annotate “Rücken” (anatomy) and Schmerzen (pain) as different words using two different spans. In other cases, texts might contain several anatomical references for a pain separated by a slash, like Rücken/Hüft und Kopfschmerzen (Back/hip and headaches) that could be annotated in a single or in separated spans.

Such situations illustrate the complexity of German clinical narratives and initially led to different interpretations between annotators. To ensure a consistent approach, we decided to annotate compound words in a single span, i.e., there is no separate annotation for anatomy and symptoms (for example headache is annotated as a single entity). In case several anatomical references were separated by a slash, the anatomy and symptoms were annotated separately.

With these new rules, the annotations were harmonized. With the newly defined annotation guidelines, the other 130 examples were annotated for the final model fine-tuning.

### 3.3 Model comparison: zero-shot & LLM vs. fine-tuned models

To identify the most suitable model for reliable symptom extraction, we compared zero-shot models—GLiNER and Mistral AI—with a fine-tuned transformer-based model (SCAI-BIO/BioGottBERT). For model validation, we used manual annotations of 150 emergency department narratives; 15% of the annotated narratives were used for model validation for all the tests.

### 3.4 Performance of the mistral AI model (baseline model)

The Mistral-Nemo-Instruct-2407 model was evaluated locally (via local API access) and showed the weakest performance across all the validation categories (see Table 5).

**Table 5:** Validation results of the GliNER model (*n* = 150 narratives).

Total	Validation metrics	Entity type	Exact	Partial	Strict
	F1	0.54	0.62	0.68	0.47
	Precision	0.55	0.63	0.69	0.48
	Recall	0.52	0.60	0.66	0.46
Symptom		Entity type	Exact	Partial	Strict
	F1	0.66	0.72	0.78	0.59
	Precision	0.71	0.77	0.84	0.63
	Recall	0.62	0.68	0.74	0.56
Negation		Entity type	Exact	Partial	Strict
	F1	0.35	0.32	0.34	0.32
	Precision	0.56	0.53	0.55	0.52
	Recall	0.25	0.23	0.25	0.23
Anatomy		Entity type	Exact	Partial	Strict
	F1	0.58	0.56	0.64	0.47
	Precision	0.52	0.50	0.57	0.42
	Recall	0.65	0.62	0.72	0.53

Symptom recognition achieved a F1 score of 0.45, with a precision of 0.5. The performance in the negation recognition was acceptable, with a precision of 0.6, while the performance in anatomy detection decreased to an F1 score of 0.16, with particularly low precision values.

### 3.5 Performance of the GLiNER model

The validation results for GLiNER are shown in Table 6.

**Table 6:** Validation results of the MISTRAL model (*n* = 150 narratives).

Total	Validation metrics	Entity type	Exact	Partial	Strict
	F1	0.38	0.28	0.38	0.25
	Precision	0.50	0.36	0.50	0.32
	Recall	0.31	0.23	0.31	0.20
Symptom		Entity type	Exact	Partial	Strict
	F1	0.45	0.32	0.42	0.29
	Precision	0.61	0.44	0.57	0.39
	Recall	0.35	0.25	0.33	0.23
Negation		Entity type	Exact	Partial	Strict
	F1	0.25	0.13	0.19	0.13
	Precision	0.60	0.31	0.46	0.31
	Recall	0.16	0.08	0.12	0.08
Anatomy		Entity type	Exact	Partial	Strict
	F1	0.16	0.07	0.25	0.07
	Precision	0.19	0.08	0.30	0.08
	Recall	0.14	0.06	0.22	0.06

While the model performed reasonably well in recognizing symptoms (F1 score: 0.66), it showed some limitations in detecting negations (F1 score: 0.35; precision: 0.56).

### 3.6 Performance of the SCAI-BIO/BioGottBERT base model

The fine-tuned SCAI-BIO/BioGottBERT base model significantly outperformed the GliNER model across all categories. It achieves the highest F1 score of 0.84 for symptom and negation recognition, demonstrating a strong balance between precision and recall (see Table 7).

**Table 7:** Validation results of the fine-tuned SCAI-BIO/BioGottBERT-based model.

Total	Validation metrics	Entity type	Exact	Partial	Strict
	F1	0.75	0.71	0.77	0.66
	Precision	0.76	0.73	0.78	0.67
	Recall	0.74	0.70	0.76	0.65
Symptom		Entity type	Exact	Partial	Strict
	F1	0.84	0.73	0.79	0.73
	Precision	0.80	0.69	0.75	0.69
	Recall	0.90	0.78	0.84	0.78
Negation		Entity type	Exact	Partial	Strict
	F1	0.84	0.78	0.81	0.78
	Precision	0.78	0.72	0.75	0.72
	Recall	0.90	0.84	0.87	0.84
Anatomy		Entity type	Exact	Partial	Strict
	F1	0.73	0.73	0.81	0.67
	Precision	0.68	0.68	0.76	0.63
	Recall	0.78	0.78	0.87	0.72

Particularly in negation handling, the model shows a substantial improvement, with an F1 score of 0.84, precision of 0.78 and recall of 0.90, making it notable more reliable in distinguishing between affirmed and negated symptoms.

### 3.7 Head-to-head evaluation of model performance

In summarizing model performance, we focused on the recognition of clinically relevant entities, particularly symptoms and negations. Since our pipeline excludes negated symptoms from further analysis, we refer to the final output as the SCAI-BIO/BioGottBERT-based-Filtneg model (SBBertFilNeg).

**Figure 1:**
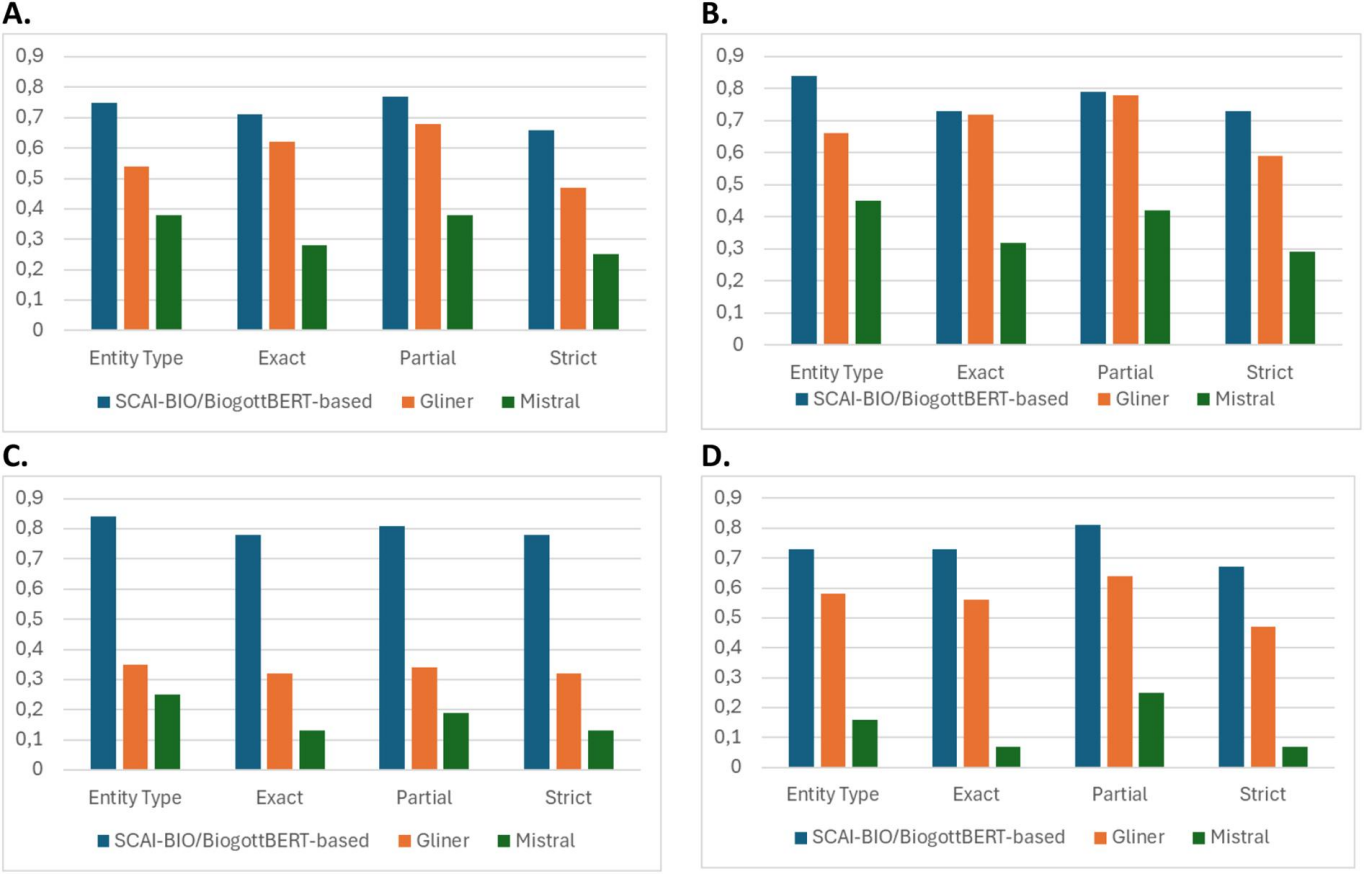
Comparison of the model F1 score of the SCAI-BIO/BioGottBERT-based (blue), GLiNER (orange) and mistral (gray) models considering the 4 different validation metrics (Table 2): validation results for total **(A)**, symptom **(B)**, negation **(C)** and anatomy **(D)**.

According to the comparative evaluation, GLiNER showed acceptable overall performance in symptom recognition but lacked reliability in detecting negations, limiting its clinical applicability. The Mistral AI model performed substantially worse across all the metrics, especially in recognizing contextual negations and anatomical references (total F1 score, see Figure 1A). In contrast, the fine-tuned SBBertFilNeg model demonstrated consistently high scores across all entity types and validation levels, confirming its suitability for structured information extraction in emergency medicine.

Thus, zero-shot models can be implemented as a first proxy for NER tasks in a clinical context but are essentially incapable of capturing subtle variations in the language, particularly complex negation forms (which are often used in German).

However, fine-tuned NLP models are more accurate in the recognition of entities in clinical texts, with a higher score with respect to zero-shot models [as visualized in Figure 1 for the four different kinds of validation methods, particularly for Symptoms (B), Negations (C) and anatomical position of symptoms (D)] and are thus more appropriate for leveraging NER tasks in clinical narratives.

Finally, this result supports other studies analyzing benchmark models in biomedicine, where the LLM-Mistral has a lower performance than other benchmark models for NER tasks [for English, see Abdul et al. (14)]. Importantly, LLM models are implemented in an on-premises implementation, and such models can perhaps perform much better when used in a cloud implementation (7).

There are two reasons that explain these results: local models generally have lower performance than online models accessed via API^9^; furthermore, LLMs are designed to predict the next words or sentences that might be needed in finding entities in narratives. Thus, these results demonstrate that LLMs have different purposes and are very limited, especially when they are applied in the biomedical field. The limitations of these models in recognizing negations in the German language are also remarkable.

## 4 Discussion

### 4.1 Context and rationale of the study

In emergency care, structured classification systems, such as ICD codes or the CEDIS, provide standardized snapshots of patient presentations. However, these predefined categories are inherently limited: they often fail to capture the nuanced, overlapping, and multisymptomatic nature of real-world clinical encounters.

Our approach instead captures the full spectrum of documented symptoms in an unbiased manner, directly from the physician's free-text notes, without reducing them to predefined categories. This enables the identification of latent symptom constellations and subgroup-specific patterns, which may remain undetected in conventional documentation frameworks.

To achieve this goal, we implemented an NLP-based extraction pipeline for unstructured clinical narratives. Given the heterogeneous language, time pressure, and varying documentation styles in the ED, this task is nontrivial. Our study demonstrates how modern NLP techniques can overcome these challenges and enable fine-grained, data-driven symptom profiling, particularly in complex patient populations such as oncology patients.

### 4.2 Methodological foundation and state of the art

Beyond textual heterogeneity and spelling errors, one key challenge lies in the diverse expression of negations, e.g., “no chest pain”, “patient denies chest pain”, or “chest pain not present”, which complicates symptom detection. This variation can greatly influence whether a symptom is interpreted as present or absent. Additionally, German and English differ substantially in grammar and lexical breadth. German's flexible syntax and compound word structures pose significant obstacles for clinical NLP (22, 23).

Moreover, German medical texts in particular have been underrepresented in NLP model training, adding to the resource gap (24).

Nevertheless, as shown below, our study demonstrates that well-selected NLP techniques, specifically NER methods, are now capable of addressing such text nuances, with limited training effort.

### 4.3 Model selection, BERT architecture and pretraining methodology

We compared three currently leading NLP model types with distinct architectures and learning strategies: for zero-shot learning, we implemented **GliNER** (18) and **Mistral-Nemo-Instruct-2407** (17); for fine-tuning, we implemented the SCAI-BIO/BioGottBERT: the fine-tuned BERT model, which was pretrained on German biomedical data (16).

BERT, as the underlying framework, was chosen for its capacity to integrate the bidirectional context and is crucial for capturing clinical semantics (15) because of its strong performance in biomedical NLP tasks, including the automated coding of electronic health records (25).

Finally, a key requirement for all the models was deployability on local servers (on-premises configuration) to meet strict data protection standards. This severe limitation affects the performance of the models: while the Mistral Nemo instruction performs below average in this study, the large Mistral model, which is accessed via API, can probably outperform and deliver better results.

### 4.4 Results of the NLP models

Our findings demonstrate that zero-shot models offer a pragmatic entry point for clinical text analysis, especially in low-resource settings, and confirm previous results that demonstrate the capability of models from the BERT family for zero-shot learning (26). However, even minimal fine-tuning with a small-annotated dataset led to marked performance improvements—particularly in negation handling. This highlights the efficiency of targeted fine-tuning and demonstrates that sustainable, locally applicable NLP pipelines are feasible without relying on large-scale generative AI models.

Furthermore, with respect to similar studies for data integration and modeling in EDs (20), we have performed much more accurate symptom extraction, which allows fine-grained symptom analysis across the population. These results lay the foundation for data-driven risk stratification and hypothesis generation in oncology patients presenting to the ED—topics we address in a dedicated follow-up study.

### 4.5 Patient-centered symptom analysis beyond predefined lists

A major strength of our approach lies in the fact that we do not rely on predefined symptom lists (11). Instead, we allow symptoms to be recorded in free text, enabling patient-centered analysis. These unstructured symptom descriptions can be organized into cluster networks and presented in a structured way to clinicians, preserving the full diversity of individual patient expression. This approach captures not only the clinical presentation but also the physician's interpretation, gender-related reporting differences, and sociocultural influences. While these aspects are inherently subjective and difficult to quantify, they are vital to understanding the true patient experience (1).

### 4.6 Clinically ready AI: efficient, local, and privacy compliant

Importantly, this analysis method was implemented with minimal computational resources, without reliance on large generative AI models. As such, the approach is well suited for local clinical infrastructures, adheres to strict data protection standards, and offers a sustainable, resource-efficient alternative to high-performance computing approaches. Our findings underscore that the future of digital healthcare may not lie in increasingly powerful AI systems but rather in intelligent, efficient modeling strategies that enhance clinical care while minimizing computational burden.

Comparative symptom frequency analysis revealed distinct clinical profiles in cancer patients compared with the general ED population, underscoring the importance of tailored symptom surveillance. The NLP-derived symptom profiles provide a solid basis for data-driven patient stratification and can be applied to enhance diagnose accuracy (27). The results of this study provide the basis for risk stratification and hypothesis generation in patients presenting to the Emergency Department, a topic of our upcoming follow-up study.

## 5 Conclusion

Symptoms recorded in unstructured ED notes contain critical clinical insights that extend beyond structured data fields such as CEDIS chief complaint systems. Our NLP pipeline captures all affirmed symptoms, preserving patient narrative complexity and individuality and enabling a patient-centered view of emergency care.

Fine-tuned, domain-specific models, particularly BERT-based approaches, outperform general LLMs and zero-shot methods in local model deployment. Even small, annotated datasets can significantly increase performance, making NLP feasible for real-world clinical use. Deployed entirely on local servers, our approach demonstrates that privacy-compliant, clinical-grade NLP is possible and implementable today.

This study bridges structured and unstructured data, enabling an enhanced layer of patient understanding. This study lays the foundation for symptom-based profiling, subgroup detection, and risk stratification in emergency oncology^10^.

Moreover, it contributes to the vision of learning from cancer centers (LCCs), where clinical care, patient-centered research, and adaptive staff training are closely intertwined. Our work supports this model by transforming routine documentation into a dynamic, data-informed feedback system for continuous learning and quality improvement.

Finally, we have demonstrated that low-resource models (zero-shot or language models trained with small data samples) have acceptable performance. One impact of using such models is that fewer resources (raw materials, energy, water, etc.) are needed, which means that sustainable and locally deployed AI models are also very effective compared to high-performance models deployed in the cloud^11^. Of course, due to the high dynamics in this area, such results and conclusions may change in the near future.

These results set the stage for hypothesis-driven stratification strategies in oncology patients presenting to the ED, which were further explored in a dedicated follow-up study.

## Data Availability

Data is available upon reasonable request to the corresponding author(s).

## Footnotes

1
https://github.com/doccano/doccano

2 See the NER dashboard at hugging face: https://huggingface.co/spaces/m42-health/clinical_ner_leaderboar

3
https://quickcreator.io/quthor_blog/bert-vs-large-language-models-differences/

4
https://www.blog.brightcoding.dev/2025/02/24/ollama-mistral-revolutionizing-local-ai-deployment-with-open-source-power/

5
https://huggingface.co/lmstudio-community/Mistral-Nemo-Instruct-2407-GGUF

6
https://github.com/urchade/GLiNER

7
https://huggingface.co/knowledgator/gliner-multitask-large-v0.5

8
https://huggingface.co/SCAI-BIO/bio-gottbert-base/tree/main

9 Using a synthetic dataset, we tested the performance of Mistral 7B in cloud computing and obtained an F1 score of 0.76; this indicates that AI implemented in the cloud is more effective than AI implemented locally.

10 A better patient understanding based on an improved data analysis of clinical narratives aligns with the SDG3, good health and wellbeing: https://www.globalgoals.org/goals/3-good-health-and-well-being/.

11 More sustainable models align with the SDG 13, climate action: https://www.globalgoals.org/goals/13-climate-action/.

## Appendix 1

The implemented prompt for the NER task using LLMs consists of three main parts:

**Part 1:** Definition of the entities that should be recognized in the narrative using the following schema:

Definition of the entities to be recognized in the text. The following entities are recognized

- Symptom (Symptom)
- Negation (Negation)
- Anatomy (Anatomie)

Dates, times, adjectives and verbs are no entities

This one is the original prompt written in German:

**Table:**

Eine Entity ist eine \"Vorerkrankung\”, \"Symptom\”, \"Negation\”, \"Anatomie\”. Daten, Zeiten, Adjektive und Verben sind keine Entitäten.

**Part 2:** Few examples with solutions are provided in the prompt, for instance:

Mr. John Doe, born on 15.04.1975, was admitted on 28.01.2025. He already has chronic hypertension, which is treated with amlodipine (5 mg daily). In addition, ibuprofen takes against back pain. On 25.01.2025, a biopsy of a tumor in the lung was performed, the results of which are still pending. There is a preliminary diagnosis of primary lung carcinoma based on CT and MRI. The patient complains of chest pain and coughing. There is no evidence of metastases or lymphatic involvement. The tumor is located in the upper right lobe of the lung. Three weeks ago, he injured himself while climbing stairs and suffered a bruise on his left thigh, which heals without complications.

Example answer:

Symptom: Chest pain |True| is a symptom of a disease
Symptom: Cough |True| is a symptom of a disease
Symptom: Weight loss |True| is a symptom of a disease
Negation: None |True| Negation of the statement

Anatomy: Upper Right Lobe of the Lung |True| describes an organ or part of the body.

The following is the original prompt written in German:

**Table:**

Herr Max Mustermann, geboren am 15.04.1975, wurde am 28.01.2025 aufgenommen. Er hat bereits eine chronische Hypertonie, die mit Amlodipin (5 mg täglich) behandelt wird. Zudem nimmt Ibuprofen gegen Rückenbeschwerden. Am 25.01.2025 erfolgte eine Biopsie eines Tumors in der Lunge, deren Ergebnisse noch ausstehen. Es liegt eine Vordiagnose auf ein primäres Lungenkarzinom vor, basierend auf CT und MRT. Der Patient klagt über Brustschmerzen und Husten. Es gibt keine Hinweise auf Metastasen oder lymphatischer Involvierung. Der Tumor befindet sich im oberen rechten Lungenlappen. Vor drei Wochen verletze er sich beim Treppensteigen und zog er sich eine Prellung des linken Oberschenkels zu, die ohne Komplikationen heilt. Beispiel Antwort: Symptom: Brustschmerzen |True| ist Symptom einer Erkrankung Symptom: Husten |True| ist Symptom einer Erkrankung Symptom: Gewichtsverlust |True| ist Symptom einer Erkrankung Negation: Keine |True| Verneinung der Aussage Anatomie: Oberer rechter Lungenlappen |True| beschreibt ein Organ oder Körperteil

**Part 3:** In the final part of the prompt the model is asked to provide the NER classification of the input text:

Use the paragraph above (see Appendix 1) to determine a list of possible entities and explain why it is either an entity or not. Return all found entities also in a Python list like this, don't add comments:

The following is the original prompt written in German:

**Table:**

Ermittele anhand des obigen Absatzes eine Liste möglicher Entities und erkläre warum es sich entweder um ein Entity handelt oder nicht. Gebe alle gefundenen Entities auch in einer Python Liste wie folgt zurück füge keine Kommentare hinzu:

The following is the result for the example provided in the second part of the prompt:

{{'word': ‘Husten’,'label': ’Symptom'}},

{{'word': ‘Brustschmerzen’,'label': ’Symptom'}},

{{'word': ‘Oberer rechter Lungenlappen’,'label': ‘Anatomie'}},

{{'word': ‘Gewichtsverlust’,'label': ’Symptom'}},

{{'word': ‘Keine’,'label': ‘Negation'}}]}}

text: {text

## References

[1] Robinson W. Epiphenomenalism. In: Zalta EN Nodelman U, editors. The Stanford Encyclopedia of Philosophy, Summer 2023. Stanford, CA: Metaphysics Research Lab, Stanford University (2023). Available online at: https://plato.stanford.edu/archives/sum2023/entries/epiphenomenalism/

[2] Tybjerg K. Medical anamnesis. Collecting and recollecting the past in medicine. Centaurus. (2023) 65(2):235–59. 10.1484/J.CNT.5.135348

[3] Raghavan P Chen JL Fosler-Lussier E Lai AM. How essential are unstructured clinical narratives and information fusion to clinical trial recruitment? AMIA Jt Summits on Transl Sci Proc. (2014) 2014:218–23. PMCID: PMC4333685. PMC4333685 25717416

[4] Kim J Podlasek A Shidara K Liu F Alaa A Bernardo D. Limitations of Large Language Models in Clinical Problem-Solving Arising from Inflexible Reasoning (2025). arXiv:2502.04381. Preprint, arXiv. 10.1038/s41598-025-22940-0 PMC12606185 41219270

[5] Munzone E Marra A Comotto F Guercio L Sangalli C Lo Cascio M Development and validation of a natural language processing algorithm for extracting clinical and pathological features of breast cancer from pathology reports. JCO Clin Cancer Inform. (2024) 8:e2400034. 10.1200/CCI.24.00034 39137368

[6] Durango MC Torres-Silva EA Orozco-Duque A. Named entity recognition in electronic health records: a methodological review. Healthc Inform Res. (2023) 29(4):286–300. 10.4258/hir.2023.29.4.286 37964451 PMC10651400

[7] Akbasli IT Birbilen AZ Teksam O. Leveraging large language models to mimic domain expert labeling in unstructured text-based electronic healthcare records in non-English languages. BMC Med Inform Decis Mak. (2025) 25(1):1. 10.1186/s12911-025-02871-6 PMC11959812 40165165

[8] Reinig I Markert K. Can current NLI systems handle German word order? Investigating language model performance on a new German challenge set of minimal pairs. In: Amblard M Breitholtz E, editors. Proceedings of the 15th International Conference on Computational Semantics. Nancy: Association for Computational Linguistics (2023). p. 1–15. https://aclanthology.org/2023.iwcs-1.1/

[9] Luo X Gandhi P Storey S Huang K. A deep language model for symptom extraction from clinical text and its application to extract COVID-19 symptoms from social media. IEEE J Biomed Health Inform. (2022) 26(4):1737–48. 10.1109/JBHI.2021.3123192 34705659 PMC9074854

[10] Marshall K Strony R Hohmuth B Vawdrey DK. New coding guidelines reduce emergency department note bloat but more work is needed. Ann Emerg Med. (2023) 82(6):713–17. 10.1016/j.annemergmed.2023.07.023 37656109

[11] Bergsneider BH Armstrong TS Conley YP Cooper B Hammer M Levine JD Symptom network analysis and unsupervised clustering of oncology patients identifies drivers of symptom burden and patient subgroups with distinct symptom patterns. Cancer Med. (2024) 13(19):e70278. 10.1002/cam4.70278 39377555 PMC11460217

[12] Corlade-Andrei M Iacobescu R-A Popa V Hauta A Nedelea P Grigorasi G Navigating emergency management of cancer patients: a retrospective study on first-time, end-stage, and other established diagnoses in a high turnover emergency county hospital. Medicina (B Aires). (2025) 61(1):1. 10.3390/medicina61010133 PMC11767032 39859115

[13] Vaswani A Shazeer N Parmar N Uszkoreit J Jones L Gomez AN Attention is all you need. Proceedings of the 31st International Conference on Neural Information Processing Systems; Red Hook, NY, USA: NIPS’17 (2017). p. 6000–10

[14] Abdul WM Pimentel MAF Salman MU Raha T Christophe C Kanithi PK Named Clinical Entity Recognition Benchmark (2024). arXiv:2410.05046. Preprint, arXiv, October 7. 10.48550/arXiv.2410.05046

[15] Devlin J Chang M-W Lee K Toutanova K. BERT: pre-training of deep bidirectional transformers for language understanding. In: Burstein J Doran C Solorio T, editors. Proceedings of the 2019 Conference of the North American Chapter of the Association for Computational Linguistics: Human Language Technologies, Volume 1 (Long and Short Papers). Minneapolis, MN: Association for Computational Linguistics (2019). p. 4171–86. 10.18653/v1/N19-1423

[16] Diaz Ochoa JG Juan G Mustafa FE Weil F Wang Y Kama K The aluminum standard: using generative artificial intelligence tools to synthesize and annotate non-structured patient data. BMC Med Inform Decis Mak. (2024) 24(1):409. 10.1186/s12911-024-02825-4 39732668 PMC11681671

[17] Jiang AQ Sablayrolles A Mensch A Bamford C Chaplot DS de las Casas D Mistral 7B (2023). arXiv:2310.06825. Preprint, arXiv. 10.48550/arXiv.2310.06825

[18] Stepanov Ihor, and Shtopko Mykhailo. 2024. GLiNER Multi-Task: Generalist Lightweight Model for Various Information Extraction Tasks. arXiv:2406.12925. Preprint, arXiv. 10.48550/arXiv.2406.12925

[19] Ashok D Lipton ZC. PromptNER: Prompting for Named Entity Recognition (2023). arXiv:2305.15444. Preprint, arXiv. 10.48550/arXiv.2305.15444

[20] Zhang X Wang Y Jiang Y Pacella CB Zhang W. Integrating structured and unstructured data for predicting emergency severity: an association and predictive study using transformer-based natural language processing models. BMC Med Inform Decis Mak. (2024) 24(1):1. 10.1186/s12911-024-02793-9 39633370 PMC11619330

[21] Tsai RT-H Wu S-H Chou W-C Lin Y-C He D Hsiang J Various criteria in the evaluation of biomedical named entity recognition. BMC Bioinformatics. (2006) 7(1):1. 10.1186/1471-2105-7-92 16504116 PMC1402329

[22] Jantscher M Gunzer F Kern R Hassler E Tschauner S Reishofer G. Information extraction from German radiological reports for general clinical text and language understanding. Sci Rep. (2023) 13(1):2353. 10.1038/s41598-023-29323-3 36759679 PMC9911592

[23] Borchert F Lohr C Modersohn L Langer T Follmann M Sachs JP GGPONC: a corpus of German medical text with rich metadata based on clinical practice guidelines. In: Holderness E Yepes AJ Lavelli A Minard A-L Pustejovsky J Rinaldi F, editors. Proceedings of the 11th International Workshop on Health Text Mining and Information Analysis. Association for Computational Linguistics (2020). p. 38–48. 10.18653/v1/2020.louhi-1.5

[24] Noll R Frischen LS Boeker M Storf H Schaaf J. Machine translation of standardised medical terminology using natural language processing: a scoping review. New Biotechnol. (2023) 77:120–29. 10.1016/j.nbt.2023.08.004 37652265

[25] Wang J Huang JX Tu X Wang J Huang AJ Laskar MTR Utilizing BERT for information retrieval: survey, applications, resources, and challenges. ACM Comput. Surv. (2024) 56(7):1–33. 10.1145/3648471

[26] Wang Y Wu L Li J Liang X Zhang M. Are the BERT family zero-shot learners? A study on their potential and limitations. Artif Intell. (2023) 322:103953. 10.1016/j.artint.2023.103953

[27] Aissaoui Ferhi L Amar MB Choubani F Bouallegue R. Enhancing diagnostic accuracy in symptom-based health checkers: a comprehensive machine learning approach with clinical vignettes and benchmarking. Front Artif Intell. (2024) 7:1397388. 10.3389/frai.2024.1397388 39421435 PMC11483353

[28] Diaz Ochoa JG Layer N Mahr J Mustafa FE Menzel CU Müller M Optimized BERT-based NLP outperforms zero-shot methods for automated symptom detection in clinical practice. medRxiv. (2025). 10.1101/2025.04.21.25326037 PMC12689901 41383383

